# Geological archive of the onset of plate tectonics

**DOI:** 10.1098/rsta.2017.0405

**Published:** 2018-10-01

**Authors:** Peter A. Cawood, Chris J. Hawkesworth, Sergei A. Pisarevsky, Bruno Dhuime, Fabio A. Capitanio, Oliver Nebel

**Affiliations:** 1 School of Earth, Atmosphere and Environment, Monash University, Melbourne, VIC 3800, Australia; 2 Department of Earth Sciences, University of St Andrews, St Andrews, Fife KY16 9AL, UK; 3 School of Earth Sciences, University of Bristol, Wills Memorial Building, Queens Road, Bristol BS8 1RJ, UK; 4 ARC Centre of Excellence for Core to Crust Fluid Systems (CCFS) and Earth Dynamics Research Group, The Institute for Geoscience Research (TIGeR), Department of Applied Geology, Curtin University, GPO Box U1987, Perth, WA 6845, Australia; 5 CNRS-UMR 5243, Géosciences Montpellier, Université de Montpellier, Montpellier, France

**Keywords:** plate tectonics, Archaean, palaeomagnetics, lithosphere, early Earth

## Abstract

Plate tectonics, involving a globally linked system of lateral motion of rigid surface plates, is a characteristic feature of our planet, but estimates of how long it has been the modus operandi of lithospheric formation and interactions range from the Hadean to the Neoproterozoic. In this paper, we review sedimentary, igneous and metamorphic proxies along with palaeomagnetic data to infer both the development of rigid lithospheric plates and their independent relative motion, and conclude that significant changes in Earth behaviour occurred in the mid- to late Archaean, between 3.2 Ga and 2.5 Ga. These data include: sedimentary rock associations inferred to have accumulated in passive continental margin settings, marking the onset of sea-floor spreading; the oldest foreland basin deposits associated with lithospheric convergence; a change from thin, new continental crust of mafic composition to thicker crust of intermediate composition, increased crustal reworking and the emplacement of potassic and peraluminous granites, indicating stabilization of the lithosphere; replacement of dome and keel structures in granite-greenstone terranes, which relate to vertical tectonics, by linear thrust imbricated belts; the commencement of temporally paired systems of intermediate and high dT/dP gradients, with the former interpreted to represent subduction to collisional settings and the latter representing possible hinterland back-arc settings or ocean plateau environments. Palaeomagnetic data from the Kaapvaal and Pilbara cratons for the interval 2780–2710 Ma and from the Superior, Kaapvaal and Kola-Karelia cratons for 2700–2440 Ma suggest significant relative movements. We consider these changes in the behaviour and character of the lithosphere to be consistent with a gestational transition from a non-plate tectonic mode, arguably with localized subduction, to the onset of sustained plate tectonics.

This article is part of a discussion meeting issue ‘Earth dynamics and the development of plate tectonics'.

“If it looks like a duck, swims like a duck, and quacks like a duck, then it probably is a duck” (paraphrase of statement by James Riley (1849–1916))

## 1. Introduction

Plate tectonics is a key feature of our planet. The generation of lithospheric plates and their interactions with mantle, atmosphere and oceans have produced the environment and resources that support the biosphere. Plate tectonics and associated feedbacks are a response to secular cooling of the Earth's interior. But heat loss also occurs through episodic processes such as the emplacement of mantle-derived magma in large igneous provinces (LIP). The relative contribution of, and the control exerted by, continuous and episodic mechanisms of heat loss may have varied through time, perhaps in response to decreasing heat flow (e.g. [1]). This uncertainty has led to debate as to how long plate tectonics has been the modus operandi of lithospheric formation and interaction with suggestions ranging from the Hadean (greater than 4 Ga) to the Neoproterozoic (less than 1 Ga) [2–5]. In significant part, these reflect differences in what might be regarded as the onset of plate tectonics, and how best to recognize plate tectonics in the geological record. A variety of non-plate tectonic modes related to evolving tectonothermal environments have been observed or proposed for other bodies in the solar system and for the early Earth ([6] and references therein). Those advocating a pre-plate tectonic regime for the early Earth generally invoke a fixed or episodically mobile lithosphere, often referred to as a stagnant-lid, but perhaps more appropriately as a single-lid. This is thought to have involved a convecting mantle egressing heat through a combination of conduction, mantle plume-focused igneous activity, and periodic catastrophic overturn of the lithosphere back to the mantle with consequent resurfacing of the solid Earth [7–10].

Uncertainty into the nature and presence of pre-plate tectonic regimes and the timing of any change in tectonic regimes relates to the incompleteness of the rock archive in deep time, differences in the criteria used to infer the existence of plate tectonic and pre-plate tectonic regimes on the early Earth (*ca* greater than 2.5 Ga), and disagreements in the significance and interpretation of available data. It is increasingly accepted, for example, that as subduction may be localized and even transient, evidence for subduction should not necessarily be taken as evidence for sustainable plate tectonics. In this paper, we therefore discuss evidence for the development of rigid lithosphere and for relative lithospheric plate motion derived from proxy data including sedimentary rock associations ascribed to divergent and convergent basin environments, granitoid associations indicative of thickening and stabilization of the lithosphere, structural features indicating a change from vertical to horizontal tectonics, P and T regimes derived from metamorphic rock associations and their tectonic settings, as well as palaeomagnetic data for substantial lateral motion of continental lithosphere blocks. We conclude that significant changes in Earth behaviour occurred in the Meso- to Neoarchaean, around 3.2–2.5 Ga, and that these changes are consistent with the transition from a pre-plate tectonic, stagnant-lid setting, in which any subduction was transient, to a regime of sustained plate tectonics, involving a linked system of convergent, divergent and strike-slip plate boundaries.

## 2. Plate tectonics: characteristics

**Figure 1.**
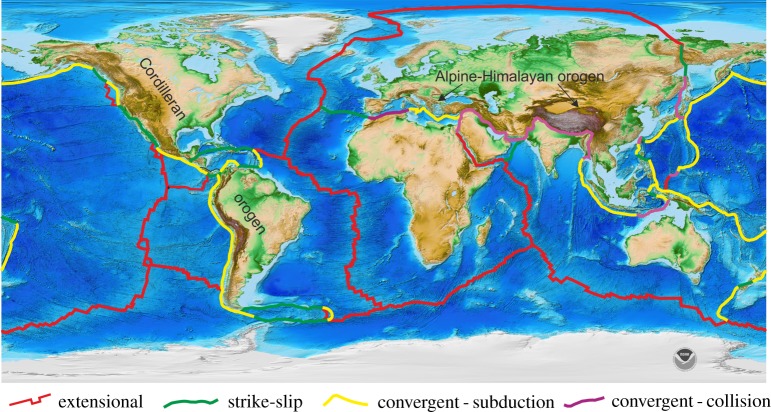
Present-day Earth global relief showing distribution of spreading, subduction, collisional and strike-slip boundaries. Global relief model based on National Oceanic and Atmospheric Administration (NOAA) ETOPO1 1 Arc-Minute Global Relief Model [11].

**Figure 2.**
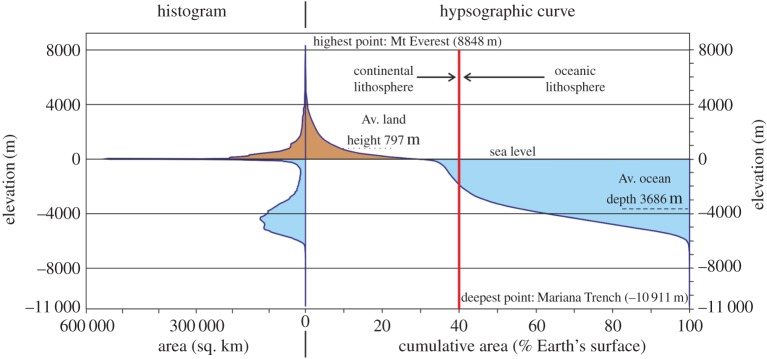
Histogram and hypsographic curve of land and ocean areas derived from ETOPO1 global relief model of Amante & Eakins [11].

On a plate tectonic Earth, the rigid outer layer, the lithosphere, is broken into tessellated fragments that are in independent horizontal motion about axes of rotation (Euler poles) at a set rate and direction (angular motion). This results in a linked system involving the divergent, convergent and strike-slip relative surface motion of rigid plates (figure 1), with deformation focused at boundary zones. The plates are made up of continental and oceanic lithospheres with contrasting chemical–physical properties [12]. Oceanic lithosphere is thin, dense with low mean elevation (largely submarine), and with new crust of largely mafic composition, whereas continental lithosphere is thicker, less dense, has higher mean elevation, and has a crustal component of more intermediate composition. The area ratio of oceanic to continental lithosphere is approximately 60 : 40 [13], with some 75% of the latter presently exposed above sea level (figure 2). Oceanic lithosphere forms at divergent plate boundaries through adiabatic decompression melting of the asthenosphere, and due to its greater overall density than the underlying asthenosphere, at least for all but the youngest lithosphere, is recycled back into the mantle at convergent plate boundaries [14,15]. The resultant slab pull is the major, on-going driving force of plate tectonics, estimated to contribute some 80% of the overall force, resulting in all oceanic lithosphere younger than 200 Ma. Fluid flux from the subducting oceanic lithosphere results in melting of the overlying mantle wedge and generation of a magmatic arc in the upper plate [16–18]. In contrast with oceanic lithosphere, continental lithosphere is buoyant with respect to the asthenosphere, resists recycling, is as old as 4 Ga, and is the archive of Earth history [19]. The bulk composition of the continental crust is similar to calc-alkaline andesite [20,21]. On the modern Earth, andesite is the characteristic rock type formed in the upper plate of convergent plate margins and constitutes the inferred major site for its formation in the past. Plate boundaries form a global, kinematically linked and dynamic system (figure 1) in which the total area of lithosphere generated at plate boundaries over medium- to long-term timescales is compensated by recycling back into the mantle, maintaining a constant-radius Earth.

The geological and geophysical manifestations of plate tectonics on the modern Earth are relatively well-studied and understood (for example, mid-ocean ridges and transform faults, rift zones, passive margins, Benioff zones, magmatic arc— trench systems, back arc basins, foreland basins, mountain ranges). However, extrapolating these established proxies of plate tectonics to the geological past is hindered by the lack of information from the oceans, increasing gaps in the geological record with increasing age, the lack of large-scale geometric constraints, and potential changes in the nature of the geological environment on the hotter early Earth, leading to uncertainty in the contribution of plate tectonics and/or non-plate tectonic processes to the long-term geological record (e.g. [5,9] and references therein). Plate tectonics is an ongoing, self-sustaining system of moving, interacting plates, and it is not always clear how to establish such a long-lived sustainable process from that preserved in the rock record.

## 3. Evidence for rigid lithosphere

**Figure 3.**
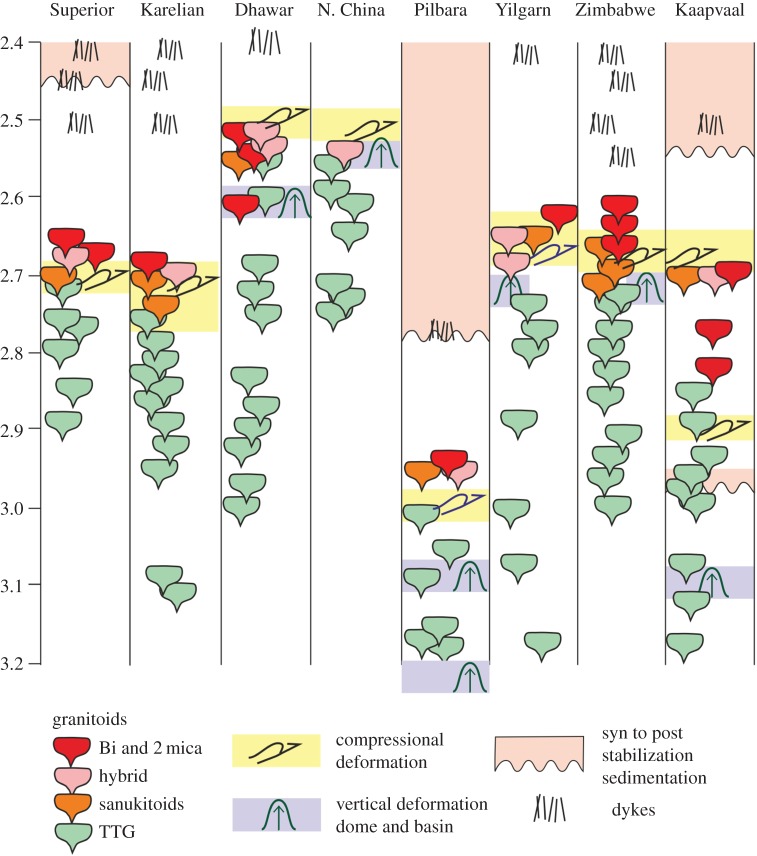
Time-space plot for the Superior, Karelian, Dhawar, North China, Pilbara, Yilgarn, Zimbabwe and Kaapvaal cratons for the period 3.2–2.4 Ga, showing the time of major pluton, mafic dyke emplacement and deformational events.

Rigid lithosphere is taken to be a necessary pre-condition for plate tectonics. Higher mantle temperatures on the early Earth lead to models of lithosphere that is impregnated with magma and has reduced viscosity and rigidity, relative to the present day. Hence it is a poor medium for stress transmission [22–24]. Geologic evidence for the existence of significant areas of rigid lithosphere on the early Earth is provided by the stabilization of cratons, the accumulation of large sedimentary basins on subsiding stable substrates, and evidence for brittle fracturing and emplacement into the cratons by rectilinear dyke swarms [25–28]. These geological features extend back to the Mesoarchean, become more prevalent in the Neoarchean and are widespread in the Proterozoic (figure 3).

Extensive sedimentary basin successions overlying stable cratons include the *ca* 2640–2055 Ma Transvaal Supergroup on the Kaapvaal craton [29,30] and the 2775–2210 Ma Mount Bruce Supergroup on the Pilbara craton [31,32]. Older and geographically smaller basin accumulations on the Kaapvaal craton extend back to *ca* 3.2–3.0 Ga [33–38] and from *ca* 3.2 to 3.0 Ga for fluvial quartz arenites on the Dharwar craton [39]. Younger, predominantly Proterozoic examples include the *ca* 2610–2120 Ma Minas Supergroup, San Francisco craton [40], 2480–2220 Ma Huronian Supergroup on the southern Superior craton [41], *ca* 2450–1900 Ga Karelian Supergroup, Baltic craton [42] and late Palaeoproterozoic to Mesoproterozoic basins of India [43].

Fracturing and dyke emplacement events within cratons extend back to 3.5 Ga for the North Atlantic craton in Greenland [44] but only become widespread both within a craton as well as globally after *ca* 3.0 Ga. The Ameralik dykes of West Greenland and the equivalent Saglek dykes in Labrador are the oldest extensive mafic intrusions cutting stable continental crust [45]. U-Pb zircon dating of the Ameralik dykes indicate they do not represent a single event but yield ages of 3.5 Ga and 3.25 Ga [44]. Younger, post-craton stabilization events include the *ca* 3.0 Ga volcanic rocks at the base of the Pongola supergroup, Kaapvaal craton [46], the 2770 Ma Black range dykes and Mount Roe Basalt, Pilbara craton [31,47,48], the 2575 Ma Great dyke, Zimbabwe craton [49,50], the *ca* 2510 Ma and 2475 Ma dykes in the Zimbabwe, Superior and Kola-Karelia cratons [50], the 2420–2410 Ma dykes in the Yilgarn, Zimbabwe, Superior and Kola-Karelia cratons [50–52] and the 2370 Ma dykes, Dharwar craton [53] (figure 3). It is very striking that dyke swarms older than these are rare, which highlights the different nature of the continental lithosphere before 3 Ga. The synchronicity of late Archean and early Palaeoproterozoic (*ca* 2.5–2.4 Ga) dyke emplacement events in the Zimbabwe, Kola-Karelia, Yilgarn and Superior cratons further suggests their spatial proximity within the inferred Superia supercontinent/supercraton [50,54].

Integrated geologic and seismologic studies of Archaean cratons indicate that they obtained their crustal and lithospheric thicknesses by the late Archaean with some intra- and intercratonic variability in thicknesses related to subsequent collision between blocks [12,55–58]. Furthermore, the secular evolution of granitoid composition from tonalite, trondhjemite and granodiorite (TTG) to K-rich metaluminous and peraluminous granites during the late Archean (3.0–2.5 Ga) as recorded in most cratons (figure 3, [59–61]) is further evidence for crustal thickening and reworking, and requires a rigid continental lithosphere by this time.

## 4. Geological evidence for plate margin interaction

Proxy data for interaction between moving lithospheric plates, whether divergent or convergent, are preserved in orogenic belts and results in distinctive patterns of geological features, which have variable long-term preservation potential. Interpretations of the Precambrian rock record must be modulated not only by an understanding of potential preservation bias, as in the skewing of sedimentary deposits towards epeiric intracontinental rather than craton margin deposits [62,63], but also by differences in parameters for the generation of rock associations. For example, the lack of vegetation resulted in braided channel environments across a range of basin depositional systems (fluvial, deltaic, tidal), carbonate accumulation mediated by changing biological controls (evolution), and the impact of secular cooling on the degree of mantle partial melting and crustal thermal regimes [62,64,65]. Even within such constraints, patterns of sedimentary, igneous, and metamorphic rock associations, and structural and metamorphic features associated with plate tectonic settings on the Phanerozoic Earth, are recognizable on the early Earth suggesting formation by similar tectonic controls.

### (a) Divergent plate boundary record

**Figure 4.**
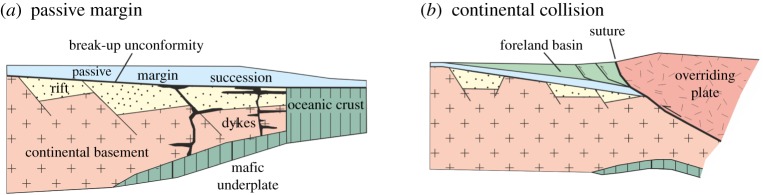
Schematic cross sections of (*a*) passive continental margin showing transition from rift to thermal subsidence phases marked by break-up unconformity and the onset of sea-floor spreading, and (*b*) continental collision resulting in loading and subsidence of lower plate and development of foreland basin succession.

The oceanic lithosphere record of plate divergence only extends back some 200 Ma, corresponding to a little over 4% of Earth's 4.56 Ga history. However, the record for continental lithospheric extension leading to break-up and sea-floor spreading extends back to the Archaean. Continental margin successions formed though lithospheric extension are divisible into rift (divergent plate) and thermal subsidence (intraplate) related basinal deposits (e.g. [66]). Rift-related facies associations consist of clastic, often terrestrial to lacustrine sedimentary units, including evaporites, with interstratified mafic and bimodal igneous rocks, and display rapid along and across strike changes in facies [67–70]. They unconformably overlie a now stabilized, pre-deformed and metamorphosed continental crust assemblage. Passive margin rock associations are composed of siliciclastic or platformal carbonates, in part reflecting palaeolatitude, with lateral continuity of facies types and constant stratigraphic thickness reflecting broad scale thermal subsidence of the rifted continental margin. The transition between rift and passive margin phases corresponds with the onset of sea-floor spreading and is marked by the break-up unconformity (figure 4*a*). Thus, the development of the earliest passive margin successions in the geological record provides a minimum age for bimodality of lithosphere into rigid continental and oceanic domains.

**Figure 5.**
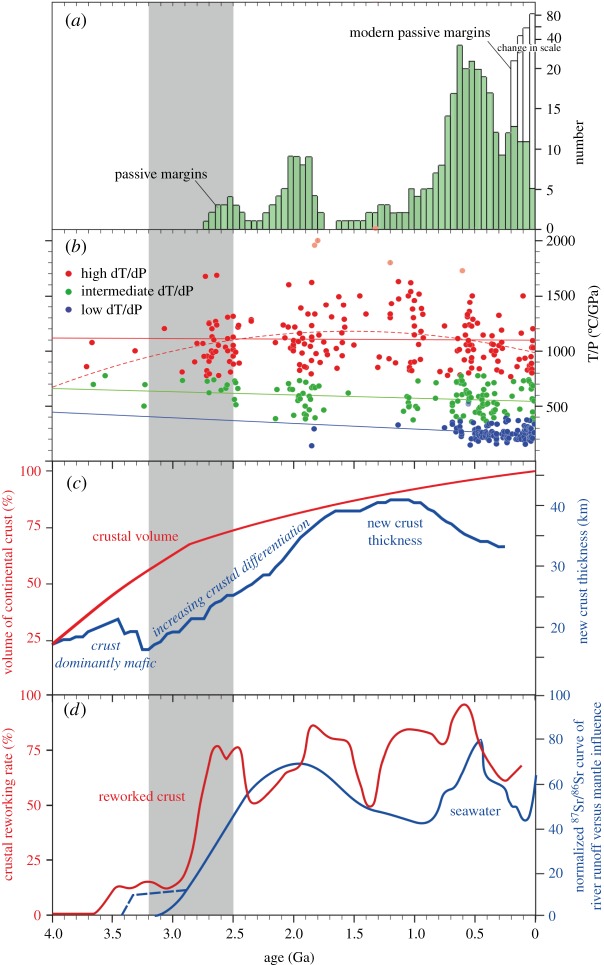
(*a*) Histogram of the ages of ancient and modern passive margins [67]. (*b*) Metamorphic thermal gradient (*T*/*P*) for 456 localities grouped as high d*T*/d*P* in red, intermediate d*T*/d*P* in green and low d*T*/d*P* in blue, and plotted against age [64]. (*c*) Crustal growth model based on Hf isotope ratios in zircons [79] and a shift in the composition of juvenile crust from mafic to more intermediate compositions accompanied by an inferred increase in crustal thickness [80]. (*d*) Moving average of *δ*18O analyses of zircon versus U-Pb ages distilled from compilation of approximately 3300 [81] and normalized seawater ^87^Sr/^86^Sr curve [82], incorporating data of Satkoski *et al*. [83,84]. Grey bar highlights age range 3.2–2.5 Ga.

The oldest ages for the rift to drift transition are preserved in the North China, Zimbabwe, Pilbara and Kaapvaal cratons and give ages in the range *ca* 2750–2500 Ma [71–76]. For example, the *ca* 2.7 Ga Fortescue Group of volcanic and sedimentary rocks can be traced for some 500 km in an east-west direction along the southern margin of the Pilbara craton, unconformably overlying basement, and inferred to record crustal extension and subsequent break-up of the craton [71,77,78]. Furthermore, the long-term temporal distribution of ancient passive margin successions is episodic and are abundant at 2100–1850 Ma and 650–500 Ma, corresponding to the assembly phases of the Nuna and Gondwana supercontinents, but are scarce during Rodinia assembly and are absent prior to 3 Ga (figure 5*a*; [67]). The purported oldest passive margin succession is in the Steep Rock carbonate platform, which unconformably overlies the tonalite basement of the Superior craton, but sediment accumulation is only poorly constrained between 3.0 and 2.8 Ga [67,85]. The temporal distribution of passive margins is in part mimicked by that of carbonates, which are generally associated with extensional tectonic settings, and are largely younger than 2800 Ma [86]. We consider the episodic temporal distribution pattern of passive margins to reflect selective preservation during the supercontinent cycle [19,87,88].

### (b) Convergent plate boundary record

Divergent lithospheric motion must be compensated by lithospheric recycling at convergence margins on a constant-radius earth. Convergent plate boundaries include subduction zones in which oceanic lithosphere is recycled back into the mantle beneath an upper plate of either oceanic (e.g. West Pacific) or continental (e.g. Andes or Japan) lithosphere, and collision zones in which opposing plates of buoyant continental lithosphere resist and ultimately terminate subduction (e.g. Alpine-Himalayan belt, figure 1). These contrasting convergent boundary types are preserved in accretionary and collisional orogens; the former generated at sites of ongoing subduction and the latter at the termination of subduction [89,90]. Each orogen type is characterized by distinctive lithotectonic assemblages, which are aligned in long linear belts parallel to the inferred plate boundary (figure 1).

### (c) Foreland basins

Foreland basins form in convergent plate settings due to lithospheric loading driven subsidence (figure 4*b*; [66,91,92]). Basin fill includes detritus from the thickened crustal pile of the overriding plate. The oldest inferred examples occur at around 3.0 Ga and include the Witwatersrand Basin, South Africa, the Cheshire Formation of the Belingwe Greenstone Belt, Zimbabwe and the Pontiac Basin, Canada. They are characterized by siliciclastic-dominated sedimentary succession, with lateral facies and thickness variations reflecting syn-sedimentary tectonic instability including disruption of the basin succession by thrust faults and/or tectonothermal events in adjoining blocks with the developing orogenic belt then acting as a sediment source. The late Mesoarchaean (3.0–2.8 Ga) Witwatersrand Basin formed during amalgamation and stabilization of the Kaapvaal craton in southern Africa, and it contains detritus derived from the bounding exhumed cratonic blocks and accreted granite-greenstone terranes (e.g. Barberton and Kimberley [26,34,38]). Facies and stratigraphic relations for the 2.65 Ga Cheshire Formation of the Belingwe Greenstone Belt are interpreted to indicate a lower carbonate succession, representing passive margin sedimentation on an older continental rift succession, and an upper siliciclastic succession that accumulated in an asymmetric basin [73]. The basin was deformed soon after sediment accumulation by north-directed thrusts and is interpreted as a foreland basin formed ahead of a northwest advancing thrust stack [93]. Foreland basins settings have also been proposed for Neoarchaean sedimentary successions (*ca* 2.7 Ga) in the Superior Province of Canada, and are unconformably overlain by molasse basin deposits marking stabilization of the craton [94,95]. Proterozoic foreland basins have been described from Africa, Australia, India and Canada [26,63,96].

### (d) Metamorphic assemblages

Metamorphic mineral assemblages provide a record of heat flow within the crust at the time of their formation, which in turn is a function of tectonic environment. A compilation by Brown & Johnson [64] of temperature, pressure, thermal gradients and age of metamorphism for mineral assemblage from some 450 localities ranging in age from Eoarchaean (less than 3800 Ma) to Cenozoic (less than 65 Ma) is divisible into three groups based on their thermal gradients (figure 5*b*): high dT/dP with thermal gradients of greater than 775°C GPa^−1^ resulting in upper amphibolite and granulite facies, and ultrahigh temperature (UHT) metamorphic rocks; intermediate dT/dP with thermal gradients of 375–775°C GPa^−1^ and producing eclogite, high pressure granulite and high pressure amphibolite facies rocks; and low dT/dP with thermal gradients of less than 375°C GPa^−1^ associated with blueschists, low temperature eclogites, and coesite and diamond facies ultrahigh-pressure (UHP) metamorphic rocks. The latter assemblages are largely restricted to Cryogenian (less than 850 Ma) and younger aged rocks [97], except for three Palaeoproterozoic (*ca* 1.8 Ga) examples [98–100] and one Mesoproterozoic (*ca* 1.14 Ga) example [101]. Brown & Johnson ([64] and references therein) argue that the intermediate and high dT/dP gradients represent temporally paired systems that commenced at the end of the Mesoarchaean (less than 2800 Ma). The intermediate thermal gradients are attributed to subduction and collisional settings, and the higher gradients with the hinterland of the overriding plate (e.g. high T back arc environments; cf. [102]) or oceanic plateaus.

Stevens & Moyen [103] describe a spatially linked paired metamorphic belt (*ca* 3.2 Ga) from the Palaeoarchaean Barberton granite-greenstone terrane. In the recent geologic past, paired metamorphic belts are related spatially as well as temporally, and the implications of paired systems only known to be linked temporally are still being debated. Low dT/dP assemblages, represented by rock types such as blueschists, are linked to the subduction of cold oceanic lithosphere, which depress geotherms, and are taken as indicative of contemporary convergent plate interaction associated with secular cooling of the mantle [3]. UHP coesite and diamond-bearing assemblages are modelled within a cooler mantle environment (less than 100°C higher than present-day mantle potential temperatures), which enables a deeper level of detachment of the oceanic plate lithosphere from the lower continental lithosphere during continent-continent collision [104].

### (e) Deformation features

The boundaries of lithospheric plates on the present-day Earth are long curvilinear features (figure 1) with stress transmitted through the rigid plates and focused at boundaries as evidenced by the distribution of earthquake foci. Coupling across convergent boundaries results in crustal thickening and stabilization forming accretionary and collisional orogens (e.g. Cordillera and Alpine-Himalayan belts, figure 1). Within these mountain belts sedimentary, deformational, metamorphic and magmatic elements are aligned parallel to the orogen reflecting the temporal and spatial integration of processes associated with initial tectonic setting (e.g. continental rifting, passive margin formation, subduction initiation) and those associated with the stabilization of these pre-existing tectonic associations into the geological archive (e.g. crustal thickening and metamorphism of plate margin assemblages during orogenesis) [89,105,106]. Long, linear accretionary and collisional orogens are recognized throughout the Phanerozoic and Proterozoic and are testimony to the operation of long, linear plate boundaries reflecting horizontal motions of plates through Wilson cycles of oceans opening and closing; for example, the largely Phanerozoic Variscan, Appalachian-Caledonian, Uralian, Terra Australis and Central Asian orogens, and the Proterozoic Cadomian, East African-Mozambique, Grenville-Sveconorwegian-Sunsas, Mazatzal-Yavapai, Svecofennian, Capricorn, Trans-Hudson, Trans-North China, Eburnean, Karelia, Akitan and Aldan orogens.

In contrast with modern orogenic belts, those preserved in Archaean cratons are small with limited along strike extent, and as a consequence the orogenic tracts lack pronounced length to width ratios. However, the original extent of the cratons has been modified by subsequent cycles of continental accretion and dispersal. For example, the Yilgarn craton consists of a series of largely linear granite-greenstone belts that were assembled through late Archaean accretionary events [107,108]. The belts have an overall north-south trend but are truncated along the northern and southern margins of the craton resulting in a present-day equidimensional aspect ratio but indicating a greater and unknown original linear extent. Thrust faults and associated asymmetric folding are features of a number of Archaean blocks (e.g. southwest Greenland, southern Africa, Yilgarn) and consistent with models involving extensive horizontal shortening [109–111] forming linear structural patterns that can be traced for hundreds if not thousands of kilometres across at least late Archaean cratons [112,113]. The Greenland example consists of a thrust stack of Meso- and Neoarchaean high-grade gneisses separated by mylonites which indicate top to the northwest sense of movement that is constrained to around 2.7 Ga on the basis of synkinematic granite sheets intruded along the mylonites [110]. Furthermore, Komiya *et al*. [114] argue on the basis of mapping in the northern part of the Isua Belt, West Greenland, for an inferred ocean floor stratigraphy of basalt, chert and turbidites repeated across strike by low-angle thrusts. They interpret this lithotectonic association to represent an accretionary complex formed at a plate boundary trench through off-scrapping of the upper crustal portion of subducted rigid oceanic lithosphere. Inferred ocean floor and trench deposits have also been mapped within the 1300 km-long accretionary orogen in the central segment of the North China Craton [115,116].

At a crustal scale, seismic reflection profiles have been used to invoke horizontal Archaean crustal shortening; for example, a profile from the southern Superior craton across the boundary between the Opatica Plutonic Belt and the Abitibi Greenstone Belt. North-dipping reflectors can be traced from near the base of the Abitibi Belt northward to 65 km below the Opatica Belt and they are interpreted to represent either a fossil subduction zone [117,118] or delaminated lower crust that formed in response to a collisional event further south [119].

Although some cratons consist of accreted linear granite-greenstone terranes, others are characterized by dome and keel structures resulting in an original equidimensional outcrop pattern. For example, the eastern Pilbara, Zimbabwe and Dharwar cratons consist of semi-circular granitoid domes flanked by low-grade synclinal belts of volcanic and volcaniclastic greenstones that dip and face away from the cores of the domes, with an overall radial mineral stretching lineation [120–123]. These features are interpreted to form through vertical, diapiric motion of granitoid bodies (figure 3) driven by density contrasts between granitic crust beneath a thick, dense and insulating greenstone cover succession [124,125]. Dome and keel structures contrast with fold and thrust belts related to horizontal motion of the lithosphere and which characterize crustal development in younger orogens. In the well-studied East Pilbara region, pluton emplacement and doming extended over hundreds of millions of years, not just across the craton as a whole but also within individual plutons [126,127]. Dome and keel structures in the Pilbara ceased around 3.1 Ga and were replaced by linear structural patterns related to horizontal tectonics [128]. The timing of this change also corresponds with a change in geochemistry from within-plate-like igneous rocks to rocks with subduction-related signatures [129]. In the Dharwar craton, India, dome and basin structures are related to lateral constructional flow in the lower crust of a hot orogen [130]. These structures formed at around 2.6 Ga [131–134], although the stratigraphic and provenance record of sedimentary units within the craton suggest a stable craton as early as 3.0 Ga [135]. Diapiric emplacement of *ca* 2.75 Ga granite gneiss into greenstone cover occurs locally in the western part of the Yilgarn craton [136]. Dome and basin structures are associated with *ca* 2.5 Ga granite-greenstone terranes in the Eastern Block of the North China craton [116,137,138], with structural analysis suggesting vertical motion of granite domes between synclinal greenstone belts [139,140].

### (f) Magmatic record of subduction

Igneous rocks have geochemical signatures related to their source composition and the nature of the melting process, and these in turn reflect the tectonic setting in which they were generated [141,142]. This is particularly well established for more mafic rock types, with subduction-related igneous rocks characterized by negative anomalies in Nb and Ta in mantle normalized minor and trace element patterns, and hence elevated Th/Nb and Th/Ta ratios [141]. Such geochemical discriminants work well in characterizing subduction zone rocks over the recent geologic past but their applicability to the early hotter Earth is less certain. This has led to both plate tectonic and non-plate tectonic models for early continental crust generation [143–147]. Moyen & Laurent [148] noted that Archaean mafic rocks are often clustered between subduction and non-subduction compositions, relative to those on the modern Earth, suggesting that true subduction systems may have been rare.

**Figure 6.**
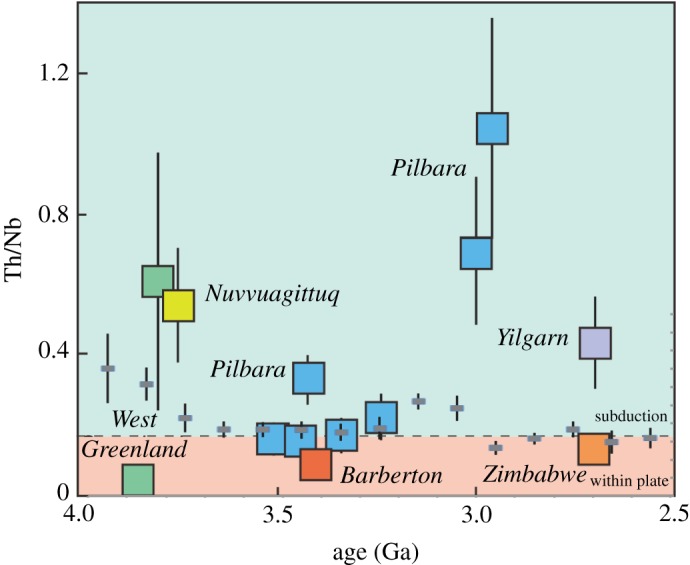
Mean Th/Nb ratios of suites of Archaean predominantly mafic rocks which are thought not to have been modified significantly by crustal contamination, after Dhuime *et al.* [153]. The green field is for elevated Th/Nb ratios, which are attributed to subduction-related processes, and the orange field is for within plate magmas. We note that in terms of mass balance it is easier to develop high Th/Nb ratios by the introduction of some from of contaminant (e.g. of pre-existing crust), than it is to develop low Th/Nb ratios by a contamination process. Data from: Barley *et al*. [154], Smithies *et al*. [150,155], O'Neil *et al*. [156], Jenner *et al*. [157,158], Puchtel *et al*. [159], Shimizu *et al*. [160] and de Joux *et al*. [161]. The small squares with different colours reflect the different locations plotted. Also plotted are the mean Th/Nb data in 100 Ma time slices (grey boxes) from a global dataset from Keller & Schoene [65].

Nonetheless, trace element ratios that are not strongly affected by variable degrees of mantle melting have been used to evaluate tectonic settings in the Archaean. Nb and Th have similar incompatibility during mantle melting and Th/Nb ratios greater than 0.2 are associated with subduction-related processes associated with the release of fluids or melts from the subducted slab [65,141]. Using a weighted bootstrapped resampling methodology of a global dataset, Keller & Schoene [65] noted little change in average Th/Nb ratios through time, and concluded that subduction-related magmatism had been ongoing throughout much of Earth's history. Other studies have recognized the presence in Archaean successions of boninitic magmas [144,149–151], and in some cases lithostratigraphic associations [152], similar to those found in more recent subduction-related settings. However, the results of detailed case studies (figure 6, and references) suggest that magmas associated with subduction and with intraplate tectonic settings were generated in different areas in the period 3.8–2.7 Ga, and also that there may be a link between dome and keel tectonics and intraplate magmatism, at least in some of these areas.

Figure 6 illustrates the tectonic settings inferred from a number of different case studies in different locations in the Archaean, and compares them with the global compilation of Th/Nb ratios from Keller & Schoene [65]. The studies depicted have used both major element and trace element proxies, and the point is not to interrogate those approaches further but to look at their outcomes and potential implications for geodynamics. Subduction-like magmatism has been recognized in West Greenland and the eastern Superior Province at approximately 3.8 Ga, and then later in the Pilbara of NW Australia at less than 3.1 Ga [129,152,157,162,163]. Magmatism with no significant subduction signal, here termed intraplate even though it may have been too early for plates to have been established, has in turn been documented in West Greenland, and in younger rocks in Barberton, Pilbara and Zimbabwe [129,158–160,164]. Moreover, in the last three examples, the intraplate geochemical signature is associated with dome and keel exposures indicating vertical tectonics. In a number of areas, subduction-like geochemical signatures extend into, and become more widespread, in younger Archaean sequences, most notably the Superior Province ([165]; but see Bédard *et al*. [166], for an alternative view [167]), the Pilbara [129], parts of the Yilgarn [168] and the North China Craton [169]. Similarly, Archaean plutonic rocks are the sodic-rich TTG suite, and are not replaced by more potassic and peraluminious plutonic rocks, typical of modern tectonic environments, until the latter part of the Archaean (figure 3; [59]).

## 5. Palaeomagnetism and lateral motion of Archaean continental blocks

Cawood *et al*. [2] and Evans & Pisarevsky [27] analysed available Archaean and Palaeoproterozoic palaeomagnetic data to test the existence of plate tectonics at those times. Both sets of analyses have been positive, but with some reservations concerning the quality and reliability of those data. For example, 2687 ± 6 Ma Mbabane Pluton pole [170] is not supported by field test and it is also located suspiciously close to younger poles. The Kaapvaal Ongeluk Lavas pole [171] has been recently re-dated [172]. On the basis of such revisions, along with new palaeomagnetic data, we have chosen a series of poles for testing the possibility of mutual lateral motions of cratonic blocks in the late Archaean to early Palaeoproterozoic. These are datasets on the oldest rocks currently available. We found three time intervals around 2680 Ma, 2505 Ma and 2440 Ma that contain coeval reliable palaeomagnetic data from the Superior and Kola-Karelian cratons with additional reliable palaeopoles also reported from the Kaapvaal craton for *ca* 2680 and 2440 Ma (table 1). There are also two coeval pairs of 2780 Ma and 2720 Ma palaeopoles from Kaapvaal and Pilbara (table 1).

**Table 1.** Palaeomagnetic data. Hiage and Lowage indicate the age range of the studied rocks. Tests refer to palaeomagnetic field tests indicating the primary remanence [173] for assessing the reliability of palaeomagnetic data. Plat, Plong and A95 refer to the measured latitude and longitude of palaeomagnetic poles and their 95% confidence circle.

Craton and age bracket	rock name	Hiage	Lowage	tests	Plat	Plong	A95	reference
2780 Ma								
Kaapvaal	Derdepoort Basalt	2787	2777	G+	−39.6	004.7	17.5	Wingate [174]
Pilbara	Black Range Dolerite Suite	2774	2770	G*+,C*+	−3.8	130.4	15.0	Evans *et al*. [175]
2720 Ma								
Kaapvaal	Westonaria Basalts	2722	2706		−17.1	047.9	18.5	Strik *et al*. [176]
Pilbara	Pilbara Flood Basalts, Packages 8–10	2721	2710		−59.1	186.3	6.1	Strik *et al*. [177]
2680 Ma								
Superior	Otto Stock Dykes and Aureole	2679	2681	C*+,R−	69.0	227.0	4.8	Pullaiah & Irving [178]
Kola-Karelia	Koitere Sanukitoids	2686	2682		−68	192.5	19.5	Mertanen & Korhonen [179]
Kaapvaal	Rykoppies Dykes	2685	2681	C+	−62.1	336.0	3.8	Lubnina *et al*. [180]
2505 Ma								
Superior	Ptamigan Mean	2507	2503		−45.3	213.0	13.8	Evans & Halls [181]
Kola-Karelia	Shalskiy Gabbronorite Dyke	2512	2504	C*+	22.7	222.1	11.5	Mertanen *et al*. [182]
2440 Ma								
Superior	Matachewan N	2449	2443	C+	52.3	239.5	2.4	Evans & Halls [181]
Kola-Karelia	Avdeev Gabbronorite, Shalskiy Dyke	2510	2441	C*+	−12.3	243.5	14.0	Mertanen *et al*. [182]
Kaapvaal	Ongeluk lava and related intrusions	2429	2423	G*+	04.1	282.9	5.3	Evans *et al*. [171];
								Gumsley *et al*. [172]

**Figure 7.**
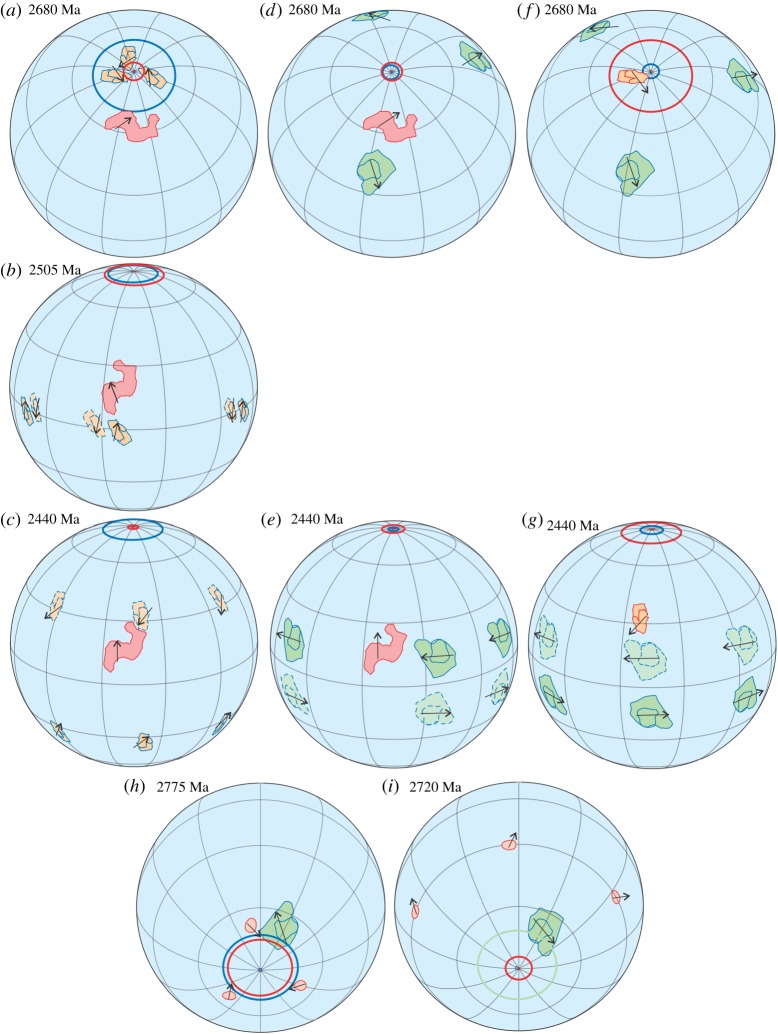
Palaeomagnetic reconstructions of Superior, Kola-Karelia and Kaapvaal cratons at 2680 Ma (*a*,*d*,*f*), 2505 Ma (*b*), 2440 Ma (*c*,*e*,*g*); of Kaapvaal and Pilbara cratons at 2775 and 2720 Ma (*h*,*i*). Arrows denote directions to the present data north. Alternative polarity option is shown with dashed outlines. See also text.

**Figure 8.**
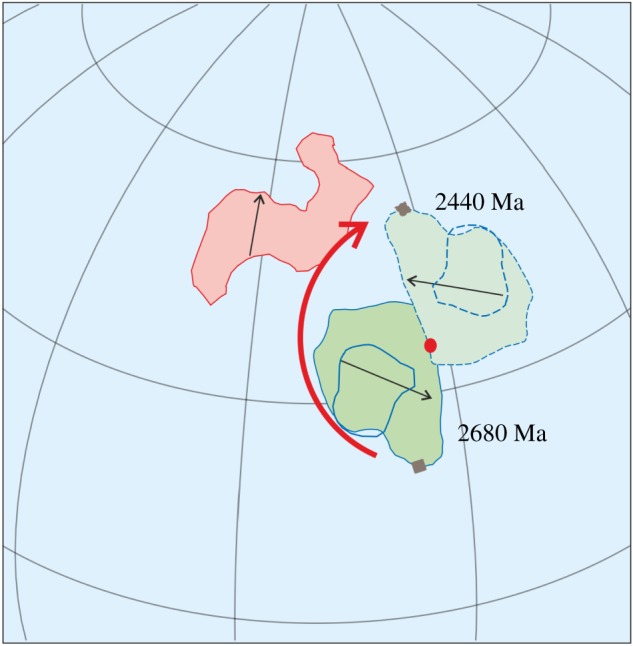
Palaeomagnetic reconstructions of Superior and Kaapvaal cratons at 2680 and 2440 Ma (in the Superior coordinates) with minimal distance between the two cratons.

Following the method used by Cawood *et al*. [2] and Evans & Pisarevsky [27], we made a series of palaeomagnetic reconstructions of: (i) Superior and Kola-Karelia at 2680 Ma, 2505 Ma and 2440 Ma (figure 7*a–c*); (ii) Superior and Kaapvaal at 2680 Ma and 2440 Ma (figure 7*d*,*e*); and (iii) Kola-Karelia and Kaapvaal at 2680 Ma and 2440 Ma (figure 7*f*,*g*). In all reconstructions, we fixed one of the two tested cratons and show schematically two polarity options (solid and dashed lines) together with longitudinal uncertainty (three possible positions along the same latitude) for the other craton. These reconstructions reveal that mutual movements of the tested cratons occurred sometime between 2680 Ma and 2440 Ma. Figure 7*h*,*i* demonstrates the same test for Pilbara and Kaapvaal for 2780 and 2720 Ma. As the palaeopoles for these two cratons suggest high latitude positions, we have shown only one polarity option. We also consider this test as positive, but admit that large circles of confidence theoretically permit reconstructions with the same mutual position of the two cratons at 2780 Ma and 2720 Ma, if we allow minimal overlap of the circles of confidence (expressed at the 95% level). Owing to longitudinal uncertainty and polarity ambiguity it is not easy to estimate the relative scale of movements of the tested cratons. However, using the reconstructions of Superior and Kaapvaal at 2680 Ma and 2440 Ma, which have the most precise palaeopoles with small circles of confidence, we estimated minimal distance of their mutual movement between 2680 and 2440 Ma. We chose the closest possible position of the two cratons at those ages and show them in Superior coordinates (figure 8). The shortest possible movement of Kaapvaal with respect to Superior is along a small circle (the pole of this circle is shown in red and the reference point in black) with the radius of about 16° by about 170° clockwise. This will require about 5100 km of movement of the reference point. All other options for these two cratons between these two time periods suggest larger distances. We conclude that there is compelling evidence for significant relative horizontal movement between cratonic blocks by the late Archaean.

## 6. Late Archaean changes in character of continental lithosphere

Sedimentological, structural, magmatic, metamorphic and palaeomagnetic datasets provide proxy evidence for lateral motion of continental lithosphere extending back until at least 3 Ga, with selected geochemical discriminant data, such as Th/Nb, inferred to represent supra-subduction zone settings at least locally as old as 3.8 Ga. Furthermore, regional and global datasets for the continental crust and its lithospheric mantle indicate significant changes in its character during the Meso- to Neoarchaean (3.2–2.5 Ga; for example, figures 3 and 5).

### (a) Continental crust composition and thickness

The continental crust has evolved from a dominantly mafic composition prior to 3.0 Ga to an andesitic composition today. For example, Tang *et al*. [183] used Ni/Co and Cr/Zn ratios in Archaean sedimentary and igneous rocks as a proxy for bulk MgO composition of the Archaean upper continental crust. It remains difficult to tie down the extent to which the rocks sampled by greenstone belt sediments are representative of the upper crust. Nonetheless, Tang *et al*. [183] concluded that this crust had an initial MgO content of *ca* 15% prior to 3 Ga, corresponding to a mafic bulk composition, but had a MgO content of *ca* 4% by 2.5 Ga, similar to modern felsic upper continental crust. Compiled records of Cr/U in terrigenous sediments suggest derivation from predominantly mafic crust prior to 3 Ga followed by a 0.5–0.7 Ga transition to crust of modern andesitic composition [184]. Similarly, statistical modelling from a database of 70 000 analyses of igneous rocks preserved within the continental crust reveal a significant change in secular trends at around 2.5 Ga inferred to involve a decrease in mantle melt fraction in basalts, and in indicators of deep crustal melting and/or fractionation (Na/K, Eu/Eu* and La/Yb ratios in felsic rocks, [185]).

The rapid shift in elemental abundances relative to the progressive decrease in mantle temperature around the Archaean–Proterozoic boundary indicates mantle temperature alone cannot be the driver for these changes [65], and are consistent with changes in processes of crust generation. Dhuime *et al*. [80] documented a progressive increase in the median Rb/Sr content of igneous rocks at the time of their extraction from the mantle, from relatively low values prior to 3 Ga rising to maximum values between 1.8 to 1.0 Ga. There is a positive correlation between Rb/Sr ratio, SiO_2_ content, and thickness of crust for the compiled dataset [80]. The changing Rb/Sr equates to SiO_2_ content and thickness of new continental crust, increasing from *ca* 48% and 20 km before 3.0 Ga to more intermediate compositions of up to *ca* 57% and 40 km thick crust by 1.8 Ga before decreasing after 1 Ga to values of 55% and 30 km towards the present (figure 5*c*). By contrast, Greber *et al*. [186], using *δ*^49^Ti isotopic composition of shales, which they argue is a proxy for SiO_2_ content of the source, noted values that are both similar to felsic rocks as well as relatively constant over the last 3.5 Ga. Thus, they proposed that felsic rocks have been a dominant component of the eroding crust throughout this timeframe, and that the source of these fractionated compositions was plate tectonics.

### (b) Continental crustal reworking, recycling and subaerial exposure

Temporal variations in U-Pb, Hf and O isotopes in detrital zircons (for example, [79,81]) have been used to assess the relative contributions of new and reworked continental crust. Global compilations of *δ*^18^O in zircons show a marked departure away from mantle values after the Archaean [81,187], which is taken to indicate significant continental crustal reworking (and increasing crustal thickness) since that time. Spencer *et al*. [81] concluded that the highest proportion of reworked crust corresponded with periods of continent/supercontinent amalgamation and the proportion of reworked crust was uniformly low prior to about 2.7 Ga (figure 5*d*). Periods of supercontinent assembly are taken to reflect periods of continental convergence, assembly and thickening, resulting in reworking of continental lithosphere. The generally low *δ*^18^O, juvenile Hf isotopic values in the Archaean implies little in the way of crustal thickening associated with collision-related orogenesis and reworking.

Dhuime *et al*. [79] used zircon isotopic data to argue for a decrease in the rate of continental growth at around 3.0 Ga. Assuming that new continental crust has been generated at a broadly constant rate since say 4 Ga, the decrease in the rate of crustal growth at approximately 3 Ga is attributed to an increase in the rates at which continental crust is destroyed—along destructive plate margins (figure 5*c*; [153]). In a similar vein, inclusions in continental sub-lithospheric mantle diamonds display a major change at around 3 Ga with the incoming of eclogitic inclusions [188]. Eclogite provides evidence of the return of the basalt protolith to the mantle, which Shirey & Richardson [188] related to the onset of subduction but could equally be achieved via any mechanism for crustal foundering, such as delamination.

Isotopic proxies of seawater composition indicate a change towards the end of the Archaean that is related to the widespread emergence of continental crust. The ^87^Sr/^86^Sr ratio of carbonates deviates from contemporaneous mantle values by 3 Ga and possibly as early as 3.2 Ga (figure 5*d*) [83,189]. Similarly, *δ*^66^Zn values and the Nd-Hf isotopic composition of banded iron formation deviate from mantle values and shifting towards values for modern seawater by around 2.7 Ga [190,191]. These observations require the emergence and erosion of a significant area of evolved crust suggesting that by around 3.2–2.7 Ga the rheology of the continental crust was sufficiently rigid to enable it to record dyke swarms, be thickened, and become emergent (cf., [192,193]). The temporal distribution of LIP is also indicative of significant crustal emergence around this timeframe [28]. If Archaean greenstone belts are LIPs, they are largely submarine [194]. The first subaerial LIPs occur around 3 Ga in the Pilbara and Kaapvaal cratons and they become widespread across all cratons by the end of the Archaean, consistent with a major period of continental thickening and stabilization [195].

### (c) Linking geological proxies with geodynamic modelling

Late Archaean changes in the bulk composition and thickness of continental crust along with increasing amounts of reworking and recycling of the crust, its widespread subaerial exposure (figure 5), and the emplacement of dyke swarms, are indicative of stabilization of the cratons. Additional expressions of this stabilization include a change from sodic TTG magmatism to K-granites and peraluminious granites [59]. These changes in granitoid composition are often co-incident with pulses of regional deformation and metamorphism followed by a termination of tectonothermal activity and isolation of the craton from subsequent tectonic events (figure 3). The timing of these changes are not synchronous but vary from craton to craton; for example, *ca* 3.7 Ga in parts of the North Atlantic, 3.0 Ga in the Pilbara, 2.9 Ga in the Amazon, 2.85 Ga in the Kaapvaal, 2.75 in the Karelia, 2.7 Ga in Superior and Yilgarn, and 2.6 Ga in Dharwar, and 2.55 Ga in North China.

Late Archaean changes in composition and character of rock associations occur within a framework of decreasing mantle potential temperature [196,197], which is associated with a change in mantle viscosity and lithospheric rigidity [192]. Although absolute values for mantle potential temperature in the Archaean are debated (e.g. [197–199]), all involve secular cooling since at least 3 Ga, which would result in lower degrees of partial melting of the mantle and less melt impregnation within the lithosphere (cf. [23]). Secular cooling impacts on mantle viscosity, which along with increasing rigidity of the lithospheric lid [200,201], will lead to an increase in the wavelength of mantle convection, which in turn feeds back into the mechanism of heat transfer [202]. Plate tectonics is associated with mantle convection of wide aspect ratios [203], whereas the hot early Earth, whether operating in a stagnant-lid or in some other mode, would have had more vigorous convection of shorter wavelength and aspect ratio beneath a relatively viscous lid impregnated by plume-related igneous activity [204–206].

**Figure 9.**
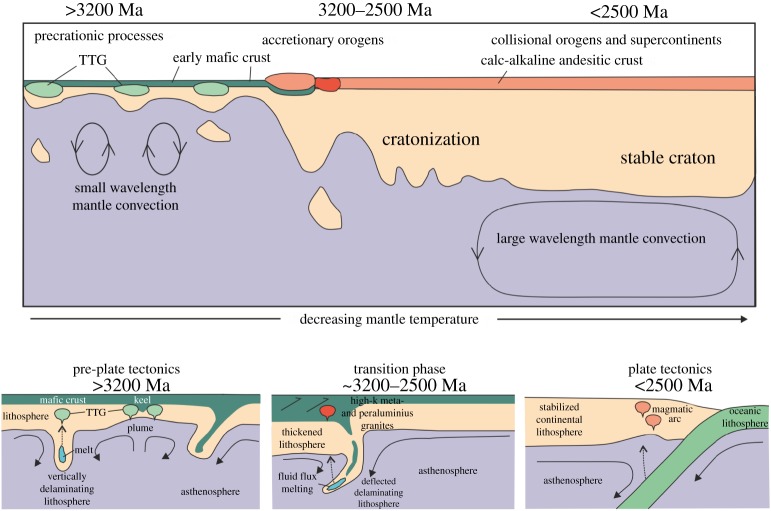
Schematic temporal evolution of the lithosphere associated with decreasing mantle temperature. Early Earth, prior to 3.2 Ga involves a non-plate tectonic regime, with magmatism characterized by a bimodal association of TTG (tonalites, trondhjemites and granodiorites) and greenstone belts. Mantle plumes are a major source of mafic magmatism. Recycling occurs through delamination and mantle convection is of relatively small wavelength. Subduction where present is transitory. Plate tectonics is envisaged to have commenced after 2.5 Ga and is associated with large aspect ratio mantle convection. Between these two tectonic regimes is a transition phase in which the lithosphere is stabilized, differentiates into oceanic and continental types, with the latter undergoing thickening enabling its emergence and erosion.

Production of continental crust, largely dominated by production of sodic TTG plutons resulted in differentiation of the lithosphere from an early largely mafic mono-lithologic crust into oceanic and continental types with contrasting density and thickness profiles. Cooling, melt extraction and dehydration of the Archaean lithosphere resulted in stabilization [55,56] of increasing volumes of continental lithosphere [79,207], which combined with their thermal blanketing effects on the convecting mantle [208,209], enable effective stress transmission through the lithosphere and its focusing at cratons' margins [210], breaking the lithosphere into plates and the formation of subduction zones [211]. Thus, we speculate that the observed changes in the geological archive in the late Archaean, in association with secular mantle cooling, corresponds with a change from a poorly mobile to a mobile lithosphere, and the reorganization of short to long wavelength mantle circulation. This marks the transition to a regime with a global pattern of linked plate boundaries, enabling sustained plate motions, and commencement of the large-scale continental assembly and dispersal. This evolving geodynamic scenario is shown schematically in figure 9.

The staggered timing between cratons in the development of different types of sedimentary basins, structural styles, metamorphic patterns and igneous rock associations and their geochemical signatures (for example, figure 3) suggest that the change from a non-plate tectonic mode to plate tectonics transitioned over some 500 to 700 Myr between *ca* 3.2 Ga and 2.5 Ga (cf. [8]). Localized and episodic subduction may have taken place prior to this transition based on geochemical signals [152,157], numerical modelling constrained from Archaean lithostratigraphic assemblages [212], modelling of major meteorite impacts [213,214], and compression around the margin of plumes [215]. Within the caveat of a fragmentary and incomplete geologic record, these inferred early pulses of subduction are not considered to form part of a globally linked system of divergent and convergent boundaries. The episodic nature of this early subduction likely reflects the infrequent nature of the driving mechanism (for example, meteorite impact), the hot nature of the mantle leading to slab breakoffs, which prevented the development of a sustained slab-pull driver for ongoing subduction, and the localized and time-limited nature of the compressive force (mantle plume) resulting in transient and non-self-sustaining subduction. In this pre-plate tectonics regime, crust formation and dehydration progressively increased the rigidity of sizeable blocks of lithosphere. Once entrained in the mantle convection pattern, the blocks could widen, migrate at larger rates, and undergo thickening and compression at their margins as they assembled, whereas other regions remained in the undeformed, poorly mobile lid tectonic mode.

## 7. Conclusion

Arguments over the timing of the initiation of plate tectonics on Earth are driven in part by differing interpretations of available spatially and temporally fragmented datasets, and through emphasis on selected data that are considered to support a particular outcome. On the modern Earth, features indicative of plate tectonics are directly observable within an established kinematic reference frame and demonstrate lateral movement of rigid plates of oceanic and continental lithosphere about Euler poles of rotation. On the early Earth, or indeed for much of the Palaeozoic and older Earth, the kinematic reference frame is lacking and oceanic lithosphere, which forms some 60% of the present-day surface, is largely lost and limited to rock associations such as ophiolites, which are themselves the source of debate as to their exact tectonic setting (compare [166,216–218]). Furthermore, interpretations of datasets are often disputed; for example, geodynamic setting derived from geochemical signatures. Thus, to date, no individual observation, or even groups of observations, provides unique undisputed evidence for plate tectonics on the early Earth, and it may be that such information is not readily preserved in the geological record. Although evidence from sedimentary, igneous and metamorphic rock associations outlined here can be related to interactions across plate boundaries, some researchers have argued that in the Archaean such features form through lithospheric movement coupled to a convecting mantle, and are not formed by a linked system of plates moving in response to ridge push or slab pull (e.g. [9]).

Our approach is to combine abductive reasoning in which we take observations from what are inevitably particular sites in the geological record, with information from more global datasets to integrate information from different scales and seek the simplest and most likely explanation. The well-known example used to illustrate this logical inference is that if something looks like a duck, swims like a duck, and quacks like a duck, then it probably is a duck. Of course, we realize that individual criteria often provide non-unique interpretations (e.g. the bill of a duck and a duck-bill platypus) but spatially and temporally linked sets of observations (bill, web feet, feathers, quaking) reduces possible alternatives. Thus, the similarity between features formed on the present day, plate tectonic Earth and those back to the late Archaean, such as passive margins, foreland basins, compressional linear structures, temporally paired metamorphic belts, and palaeomagnetically constrained relative motion of lithospheric blocks, as well as the temporal continuity of these features across this time period, validates our interpretation that they formed in a mobile lid regime related to plate tectonics. The time at which individual features appear in the geological record vary, and although this in part reflects the vagaries of preservation, they generally start to appear in the Mesoarchaean and are widespread across most cratons by the end of the Neoarchaean. This increasingly widespread distribution through the Archaean, as well as their noted similarity to features observed on a modern day plate tectonic Earth, suggest they are not isolated features related to non-plate tectonic or episodic plate tectonic processes but record the parturition of a linked system of lithospheric plates.

Recognition of features considered to be characteristic of subduction, whether they be in the Hadean, Archaean or Proterozoic and younger successions, does not directly constrain the start of plate tectonics; subduction does not in and of itself equate to plate tectonics. This is because subduction, at least in earlier parts of Earth history, was likely episodic [213,215,219]. Thus, the key issue in defining the start of plate tectonics is not in establishing when subduction first appeared on Earth, although that is a minimum prerequisite, but rather when convergent along with divergent boundaries delineated the edges of a globally linked system of plates. Unfortunately, this concept of a globally linked system can only be established in the post-Pangaea Earth in which ocean floor is preserved, enabling a kinematic reference frame of plate interaction to be established. Recent attempts have been made at full-plate global palaeogeographic models for pre-Pangaean times [220–223]. Nevertheless, when plate tectonics initiated on a pre-Pangaea Earth cannot be unequivocally proven specific proposals can be falsified [224]. The challenge for those proposing a birthdate for plate tectonics is not more evidence for subduction at specific time or place but rather to show that it was sustained and part of a global system of plate boundary interaction. We consider that the progressive development of geological proxies of rigid lithosphere and for convergent and divergent plate interaction across all the major cratons by the late Archaean is the expression of the formation of such a linked system.

In summary, we consider changes in the behaviour and character of the lithosphere in the later parts of the Archaean to be consistent with a gestational transition from a non-plate tectonic mode to the birth of plates, followed by sustained plate tectonics. We emphasize the need to avoid arguments built on observations from specific regions or data types, not least because of the difficulties in establishing the wider significance of localized datasets in highly heterogeneous material, such as the continental crust. Such information is important but it must be part of a multicomponent analysis to build geologically constrained and integrated global datasets. For it is only through the integration of information from different scales that Earth-wide linked evolutionary trends can be established, and those remain the prerequisite in recognizing tectonic modes of lithospheric plate interaction.

## Data accessibility

This article has no additional data.
